# Peroxisome Proliferator-Activated Receptor -**β**/**δ**, -**γ** Agonists and Resveratrol Modulate Hypoxia Induced Changes in Nuclear Receptor Activators of Muscle Oxidative Metabolism

**DOI:** 10.1155/2010/129173

**Published:** 2010-11-24

**Authors:** Timothy R. H. Regnault, Lin Zhao, Jacky S. S. Chiu, Stephanie K. Gottheil, Allison Foran, Siu-Pok Yee

**Affiliations:** ^1^Children's Health Research Institute and Lawson Research Institute, Department of Obstetrics and Gynecology, University of Western Ontario, London, ON, Canada N6A 5C1; ^2^Department of Physiology and Pharmacology, University of Western Ontario, London, ON, Canada N6A 5C1; ^3^Department of Genetics and Developmental Biology, University of Connecticut Health Center, Farmington, CT 06030-3301, USA

## Abstract

PPAR-*α*, PPAR-*β*, and PPAR-*γ*, and RXR in conjunction with PGC-1*α* and SIRT1, activate oxidative metabolism genes determining insulin sensitivity. In utero, hypoxia is commonly observed in Intrauterine Growth Restriction (IUGR), and reduced insulin sensitivity is often observed in these infants as adults. We sought to investigate how changes in oxygen tension might directly impact muscle PPAR regulation of oxidative genes. Following eight days in culture at 1% oxygen, C_2_C_12_ muscle myoblasts displayed a reduction of PGC-1*α*, PPAR-*α*, and RXR-*α* mRNA, as well as CPT-1b and UCP-2 mRNA. SIRT1 and PGC-1*α* protein was reduced, and PPAR-*γ* protein increased. The addition of a PPAR-*β* agonist (L165,041) for the final 24 hours of 1% treatment resulted in increased levels of UCP-2 mRNA and protein whereas Rosiglitazone induced SIRT1, PGC-1*α*, RXR-*α*, PPAR-*α*, CPT-1b, and UCP-2 mRNA and SIRT1 protein. Under hypoxia, Resveratrol induced SIRT1, RXR-*α*, PPAR-*α* mRNA, and PPAR-*γ* and UCP-2 protein. These findings demonstrate that hypoxia alters the components of the PPAR pathway involved in muscle fatty acid oxidative gene transcription and translation. These results have implications for understanding selective hypoxia adaptation and how it might impact long-term muscle oxidative metabolism and insulin sensitivity.

## 1. Introduction

Epidemiological studies highlight an association between low birth weight (LBW) and intrauterine growth restriction (IUGR) and an increased risk of metabolic syndrome, or Syndrome X, in postnatal life [1] as a result of the early development of insulin-resistance [2, 3]. Reduced insulin sensitivity, or insulin resistance, is present before overt features of the metabolic syndrome are apparent and is believed to be a critical pathophysiological event early in the adult disease process [4]. Insulin sensitivity is primarily determined by the responsiveness of skeletal muscle to insulin, since up to 90% of insulin stimulated glucose uptake takes place in this tissue. In insulin-resistant individuals, skeletal muscle typically displays altered fatty acid transport and oxidation, intramyocellular lipid accumulation, and reduced mitochondrial oxygen uptake [5, 6]. The nuclear receptor family, the Peroxisome Proliferator-Activated Receptors (PPARs) PPAR-*α*, PPAR-*β*/*δ*, and PPAR-*γ*, the PPARs' obligatory cotranscription factor, Retinoid X Receptor (RXR), and the transcriptional coactivator PPAR-*γ*, coactivator-1*α* (PGC-1*α*), are considered essential in generating normal skeletal muscle fiber type distribution, fatty acid oxidative metabolism, and insulin sensitivity [7–10]. Additionally, the sirtuin, SIRT1, has been proposed to lie at the center of a regulatory loop regulating the actions of PGC-1*α* and the PPARs, ultimately controlling muscle fatty acid oxidation [11, 12].

PGC-1*α* levels are directly correlated with overall oxidative capacity, maintaining a high number of active mitochondria and oxidative proteins, thereby increasing insulin sensitivity [13]. Reduced skeletal muscle PGC-1*α* has been linked to the development of insulin resistance and Type II diabetes [14]. Muscle PGC-1*α* expression is dependent upon PPAR-*β*/*δ* activity [9] and together with PPAR-*α*, directly and indirectly, regulate genes involved in fatty acid transport and oxidation [15–17]. Two of particular importance are carnitine palmitoyltransferase 1 (CPT-1b), involved in the mitochondrial transfer and oxidation of long-chain fatty acids [18], and the uncoupling protein (UCP-1, -2, and -3) family responsible for regulating mitochondrial electron flux and augmentation of mitochondrial fatty acid oxidation [19, 20]. Altered expression of these genes is directly correlated with insulin insensitivity, through impaired fatty acid oxidation, intramyocellular lipid accumulation, and mitochondrial damage [21–23]. 

Potential alterations to these above signaling pathways have been highlighted in the development of insulin resistance in IUGR studies [2, 3]. Low birth weight and IUGR babies display significant differences in measures of insulin sensitivity as early as 2 and 7 years of age [2, 4] and clinical outcomes consistent with peripheral insulin resistance in early adulthood [3, 24]. Further, studies in animal IUGR models report altered fetal and downstream genes involved in muscle fatty acid oxidation, such as CPT-1b and the UCP family [25], and permanent alterations in body composition, including increased adiposity [26]. These studies suggest that *in utero* programmed alterations in muscle oxidative metabolism likely manifest themselves in postnatal life.

Near term, IUGR pregnancies commonly display reductions in oxygen and other nutrient supply to the fetus [27, 28], as a result of a chronic failure in placental gas and nutrient (glucose and amino acids) exchange and function (i.e., placental insufficiency), with a corresponding reduced fetal growth. Studies concerning the effects of global placental insufficiency upon fetal muscle development and possible postnatal outcome are emerging, though direct effects of individual components of placental insufficiency, such as hypoxia, are lacking. Hypoxia is a critical regulator of fetal growth, independent of other nutrients [29, 30], and molecular oxygen plays a critical role in energy homeostasis, providing cues for modulation of gene expression. Further, prenatal influences, such as *in utero* hypoxia leading to reduced tissue oxygen supply, have been suggested to be as important as genetics and life style factors in contributing to the current “epidemic” of adult obesity and type-2 diabetes, or the metabolic syndrome [1]. However, the effects of hypoxia upon the SIRT1/PGC-1*α*/PPAR signaling pathway and downstream target genes are not well characterized. Further, enhancing muscle insulin sensitivity in human and animal models of obesity and diabetes, through the use of insulin sensitizers, such as PPAR-*β*/*δ* agonists, and the thiazolidinediones and SIRT1 modulators, such as Resveratrol, has been successful in restoring skeletal mitochondrial regulation and insulin sensitivity [31–33]. Their effects in situations of hypoxia-induced alterations upon the machinery associated with regulating insulin sensitivity are not documented. The present study is aimed to identify specific effects of changes in oxygen tension upon the PPARs, their regulators as well as downstream genes, and to investigate the regulatory programs activated by the PPAR-*β* agonist, L165041, the PPAR-*γ* agonist, Rosiglitazone, and the positive SIRT1 modulator, Resveratrol, in reduced oxygen tension environments.

## 2. Methods and Materials

### 2.1. Experimental Design

 C_2_C_12_ myoblast cell line was purchased from ATCC (Manassas, VA). The C_2_C_12_ cell line is well established for the study of PGC-1/PPAR interactions as well as oxidative capacity [34, 35] and has been previously utilized in hypoxic studies [36, 37]. Cells were cultured in DMEM supplemented with 10% fetal bovine serum to approximately 70% confluence prior to passage or differentiation induction. C_2_C_12_ cells are induced to differentiate with DMEM supplemented with 2% adult horse serum (DM). For hypoxia studies, cells were placed in one of two (5% or 1% oxygen) reduced oxygen regimes for up to eight days with medium change every 48 hours. Control samples were incubated at 21% oxygen. Hypoxia was attained by placing appropriate cultures in an anaerobic incubator (Modular Incubator Chamber (MIC-101) Billups-Rothberg, Del Mar, CA) flushed and filled with a predetermined oxygen mixture, either 1% or 5% O_2_ (with 5% CO_2_, balance N_2_). The 5% and 1% oxygen regimes were selected as being representative of fetal arterial oxygenation in the normal growth situation (5% ~38 Torr) and in the hypoxic fetal growth restricted situation (1% ~8 Torr) [28, 38]. While *in vivo* fetal muscle cell tension would be at lower concentrations, the use of relative concentrations is deemed to be suitable to highlight differential effects of altered oxygen tension [36]. Cells were harvested just prior to treatment or day zero (D0), and then at two (D2), five (D5), and eight (D8) days after the initiation of specific oxygen regimes for mRNA and protein analyses.

Additional experiments were conducted in which after seven days in set oxygen regimes; differentiated myotubes were treated with agonists for PPAR-*β* (L-165041; 10 *μ*M, EMD Biosciences, Germany) [39], PPAR-*γ* (Rosiglitozone; 10 *μ*M, Cayman Chemical) [34], the SIRT1 activator, Resveratrol (50 *μ*M, EMD Biosciences) [40], or the vehicle, DMSO. Agonist and modulator treatment concentrations were determined according to previous reports with C_2_C_12_ preparations [34, 39, 40]. Cells were then returned to their respective control (21% oxygen) or hypoxic (5% or 1% oxygen) conditions, and mRNA and protein samples were harvested 24 hours later.

### 2.2. Preparation of RNA and Real-Time PCR Procedures

Total cell lysates was collected for relative mRNA quantification. Total RNA was prepared with TRIzol reagent, and cDNAs were generated using Superscript (Life Technologies, Inc. Burlington, Ontario) and oligo dT primer. Real-time PCRs were performed using 20 ng of diluted cDNA, 200 nM of each primer, and SYBR Green PCRmix (BioRad, Hercules, CA, USA)) on the CFX384 real-time PCR detection system (Bio-Rad). A relative standard curve of pooled C_2_C_12_ cDNA was generated (six standards prepared as 4-fold serial dilutions) and used for quantification of unknown sample expression. Results were adjusted to the relative level of Ribosomal Protein L7 (RL7) mRNA and expressed relative to the average of the control group for each gene. Specific primer sets and PCR conditions for mouse LOX, SIRT1, PGC-1*α*, RXR-*α*, PPAR-*α*, *β*/*δ*, -*γ*, CPT-1b, and UCP-2 are detailed in Table 1, and specific PCR products were sequenced to confirm their identity. Target cDNA levels were quantified utilizing iQ SYBR Green Master Mix (Bio-Rad) according to the manufacturer's instructions. All results were normalized to RL7 RNA (Table 1). The relative expression was shown as a relative fold change of the target gene to zero using the Pfaffl equation [41].

### 2.3. Western Blot Analysis

Total cell lysates were collected for protein determination. Protein quantity was determined using a detergent compatible protein assay kit (Bio-Rad). Twenty micrograms of total protein were mixed with 4x sample buffer, boiled for one minute and loaded onto a 4%–12% SDS-PAGE gradient gel. After the proteins were sufficiently separated, they were transferred onto a PVDF membrane (GE Healthcare, Buckinghamshire, UK). Western blots were conducted using antibodies purchased from Santa Cruz Biotechnology (Santa Cruz, CA, USA) to determine the levels of SIRT1 (SC-15404, 1 : 200) or PGC-1*α* (SC-13067, 1 : 200) or PPAR-*γ* (SC-7196, 1 : 200) and from Abcam (Cambridge, MA, USA) to determine UCP-2 (Ab67241 (1 : 500), with an overnight incubation at 4°C. Blots were incubated for one hour with a horseradish peroxidase conjugated donkey antirabbit secondary antibody (711-035-152, 1 : 10,000; Jackson ImmunoResearch Laboratories, Inc. West Grove, PA), and specific proteins were detected using ECL chemiluminescence substrate (Thermo Fisher Scientific, Rockford, IL, USA). For a loading control, blots were reprobed using a polyclonal antibody against *β*-tubulin (ab-5046, 1 : 10,000). Western blot analysis were performed by standard methods using enhanced chemiluminescence, as per manufacturers instructions (Thermo Fisher Scientific) and quantification carried out using Quantity One software (Bio-Rad).

### 2.4. Statistical Analysis

For the 8-day culture experiments, fold differences from zero for the mRNA of the gene of interest were generated and normalized to RL7 mRNA and the Day 0 set as 1. Protein data were normalized on the respective *β*-tubulin sample and the Day 0 sample was set as 1. Normalcy of the mRNA and protein data were tested with a two-tailed F test and then analyzed using One Way ANOVA with a Tukey posttest. Data are presented as mean values ± SEM and represent 3–5 independent experiments and a *P*-value < .05 was considered statistically significant. For the PPAR-*β*/*δ*, -*γ* agonist and Resveratrol experiments, fold differences of relative expression of the specific gene of interest mRNA normalized to RL7 mRNA were reported. Within different oxygen regimes the effects of treatments (L165,041, Rosiglitazone, and Resveratrol) were evaluated by Student's *t*-test, and are presented as mean values ± SEM and represent 3–5 independent experiments. A *P*-value < .05 was considered statistically significant.

## 3. Results

### 3.1. Markers of Reduced Oxygen Tension and PPAR Expression

**Figure 1:**
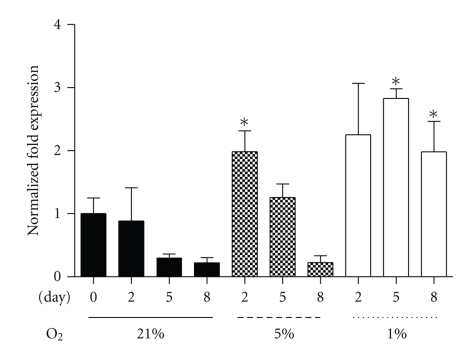
LOX mRNA in control (21% oxygen), 5%, or 1% oxygen at collection points Day 0, 2, 5, and 8. Values represent the mean of 3-4 experiments. Mean values ± SEM. ANOVA, ^*∗*^significant between control time point and corresponding hypoxic time point (*P* < .05).

To confirm that the differentiated myotubes were experiencing hypoxic conditions, expression of lysyl oxidase (LOX) mRNA, an HIF-1*α*-induced molecular marker [42] was determined. Under 1% oxygen, LOX mRNA was significantly increased over control at Day 5 (D5) and Day 8 (D8) (Figure 1). Interestingly, while LOX levels appeared to remain elevated at D8 of 1% culture, LOX mRNA declined to control levels in the 5% culture by D8. These data confirm the existence of a prolonged hypoxic environment in the 1% culture system, with HIF-1*α* induction of target genes such as LOX occurring, though a differential response was observed with respect to the 5% treatment.

**Figure 2:**
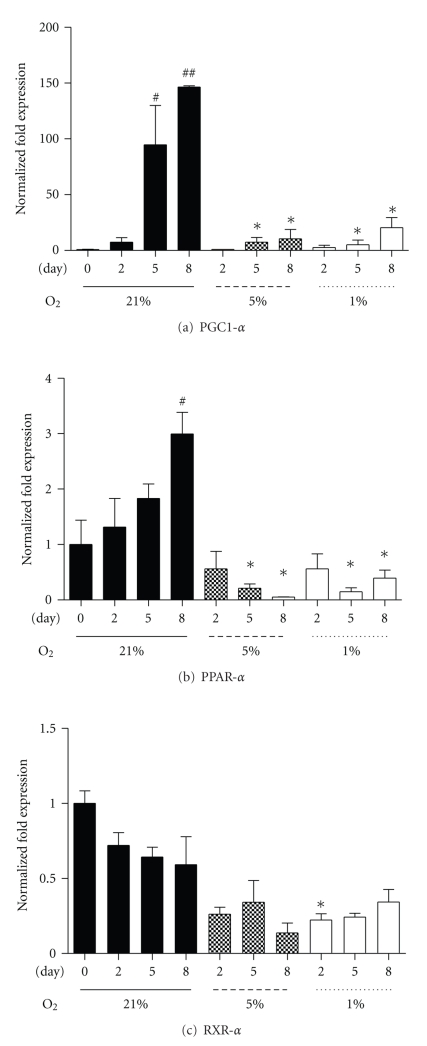
Fold changes in PGC-1*α* (a), PPAR-*α* (b), and RXR-*α* (c) mRNA at Day 0, 2, 5, and 8 under three oxygen regimes: control 21%, 5%, and 1%. Values represent the mean of 4-5 experiments. Mean values ± SEM. ANOVA, ^#^ and ^##^significant from Day 0 within 21% oxygen (*P* < .05 and *P* < .01); *significant between respective 21% oxygen time point and corresponding hypoxic time point (*P* < .05).

The PPARs, the PPARs' obligatory cotranscription factor, RXR*α*, and PGC-1*α*, are considered essential in promoting normal insulin sensitivity through regulating oxidative genes [15]. Under 21% oxygen, PGC-1*α* and PPAR-*α* mRNA rose whereas RXR-*α* remained unchanged. However, PGC-1*α* and PPAR-*α* mRNA were significantly reduced in the 1% and 5% oxygen compared to control time points by D5 (Figures 2(a) and 2(b)). RXR*α* mRNA levels remained constant at 21% over the 8-day regime (Figure 2(c)), while levels were similarly reduced as observed for PGC-1*α* and PPAR-*α*, compared to their respective control day levels (Figure 2(c)). Conversely, SIRT1 and PPAR-*β* and -*γ* mRNA were unaltered by oxygenation level (data not shown).

**Figure 3:**
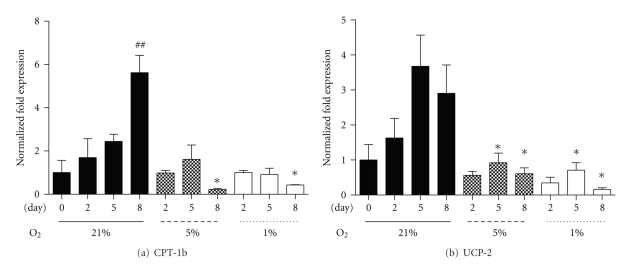
Fold changes in CPT-1b (a) and UCP-2 (b) mRNA at collection points Day 0, 2, 5, and 8 under three oxygen regimes: control 21%, 5%, and 1%. Values represent the mean of 4-5 experiments. Mean values ± SEM. ANOVA, ^##^significant from Day 0 within 21% oxygen (*P* < .01); *significant between respective 21% oxygen time point and corresponding hypoxic time point (*P* < .05).

PPAR target genes include the regulators of muscle fatty acid metabolism, such as CPT-1b and UCP-2 [15]. Similar to the rise in PGC-1*α* and PPAR-*α* mRNA over the eight-day culture under 21% oxygen, CPT-1b and UCP-2 mRNA also increased. Though under both hypoxic regimes (5% and 1%), CPT-1b mRNA was significantly reduced at D8 (Figure 3(a)), and UCP-2 mRNA was reduced at D5 and D8 (Figure 3(b)). These data show that under hypoxic conditions, PPAR target mRNAs are reduced during myoblast differentiation.

### 3.2. Hypoxia Alters SIRT1, PGC-1*α*, and PPAR-*γ* Protein

**Figure 4:**
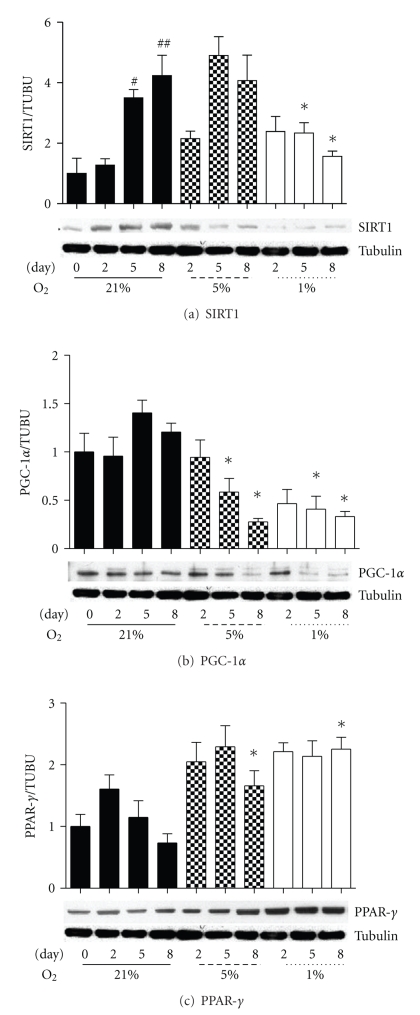
Protein level of SIRT1 (a), PGC-1*α* (b), and PPAR-*γ* (c) normalized to *β*-tubulin at Day 0, 2, 5, and 8 under three oxygen regimes: control 21%, 5%, and 1%. Values represent the mean of 4-5 experiments. Mean values ± SEM. Representative blots are shown. ANOVA, ^#^ and ^##^significant from Day 0 within 21% oxygen (*P* < .05 and *P* < .01), *significant between respective 21% oxygen time point and corresponding hypoxic time point (*P* < .05).

 A major regulator of PGC-1*α* activity is the deacetylation enzyme SIRT1 [12]. Under control 21% oxygen, SIRT1 protein level rose significantly over the period of over the 8 days of culture, while PGC-1*α* protein level was consistent. However, under 1% oxygen SIRT1 protein was significantly reduced compared to control at the D5 and D8 collection points (Figure 4(a)), despite unaltered mRNA expression, suggesting a hypoxia-induced posttranslational modification of SIRT1.

In addition, SIRT1 regulates another pathway of cellular energy metabolism, controlling PPAR-*γ* which plays a pivotal role in systemic lipid and glucose homeostasis [7]. SIRT1 represses PPAR-*γ* activity, promoting mobilization of fatty acids and decreasing fat accumulation [43], and PPAR-*γ* represses SIRT1 [44]. In conjunction with decreased SIRT1 protein, PPAR-*γ* protein was increased at both 5% and 1% oxygen, and it was significantly elevated in both hypoxic treatments at D8 (Figure 4(c)). These results suggest that under hypoxia a reduced SIRT1 protein level occurs in association with increased PPAR-*γ* protein levels.

### 3.3. PPAR-*β*, PPAR-*γ*, and SIRT1 Modulators Alter Hypoxia-Induced Alterations

**Figure 5:**
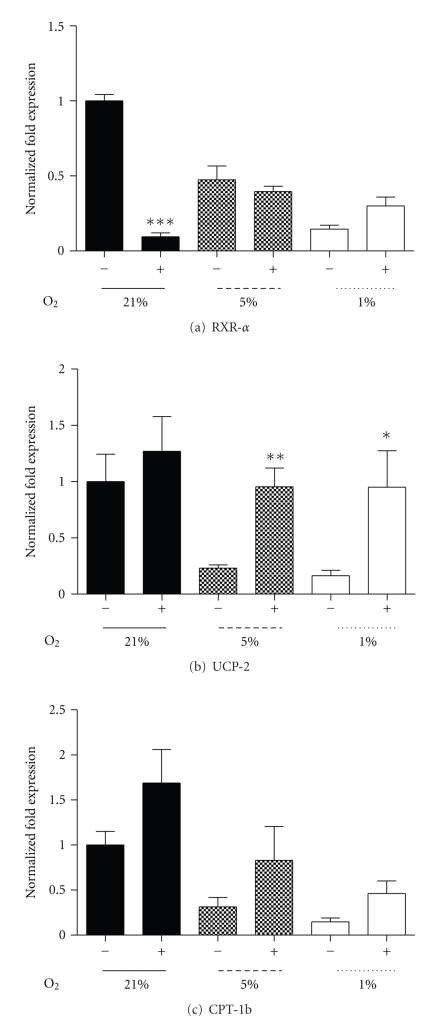
The effect of PPAR-*β*/*δ* agonist (L165,041, 10 *μ*M) treatment for 24 hours, following 7 days of differentiation under relative oxygen regimes, 21%, 5%, and 1%. Fold expression of RXR-*α* (a), UCP-2 (b), and CPT-1b (c) mRNA with (+) and without (−) L165,041. Values represent the mean of 4-5 experiments. Mean values ± SEM. The effect of L165,041 within each oxygen regime was evaluated by Students *t*-test, *P < .05, **P < .01, ***P < .001.

The administration of the PPAR*β*/*δ* agonists (GW501516 and L165041) has been shown to promote skeletal muscle fatty acid oxidation and improve insulin sensitivity in situations of high-fat diets [39, 45]. Consistent with the D8 hypoxic culture data, RXR-*α* was reduced (Figures 2(a) and 5(a)), and incubation of C_2_C_12_ myotubes with L165,041 (10 *μ*M) for 24 hours at D7 post differentiation resulted in a significant suppression of RXR-*α* mRNA under 21% oxygen (Figure 5(a)). There were no significant L165,041 effects upon SIRT1, PGC-1*α*, PPAR-*α*, or PPAR-*β*/*δ* mRNA though there were trends for increased RXR-*α* (Figure 5(a)) and PPAR-*γ* mRNA at 1% with L165,041 (*P* < .08 and *P* < .1, resp.). L165,041 stimulated increased expression of UCP-2 mRNA at both reduced oxygen treatments (Figure 5(b)). Under 5% or 1% oxygen, CPT-1b mRNA was increased, though this was not significant (Figure 5(c)). Furthermore, protein content for SIRT1 was decreased and PPAR-*γ* elevated, as previously demonstrated under hypoxia. However, treatment with L165,041 elicited no changes in protein at 21% and 5% oxygen, while UCP-2 protein was elevated at 1% oxygen when treated with L165,041 (*P* < .06).

**Figure 6:**
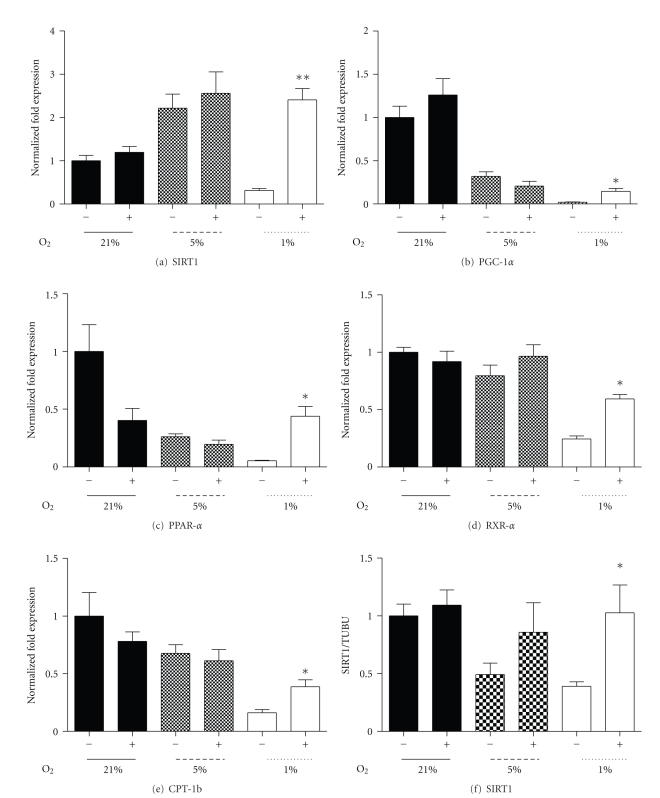
The effect of PPAR-*γ* agonist (Rosiglitazone, 10 *μ*M) treatment for 24 hours, following 7 days of differentiation under relative oxygen regimes, 21%, 5%, and 1%. Fold expression of SIRT1 (a), PGC-1*α* (b), PPAR-*α* (c), RXR-*α* (d), CPT-1b (e) mRNA, and SIRT1 protein (f) with (+) and without (−) Rosiglitazone. Values represent the mean of 3-4 experiments. Mean values ± SEM. The effect of Rosiglitazone within each oxygen regime was evaluated by Students *t*-test within oxygen regime, *P < .05, **P < .01.

PPAR-*γ* stimulation, through the induction of (PGC)-1*α*, promotes mitochondrial biogenesis and insulin sensitivity in several peripheral tissues including muscle tissues [13, 46]. Following seven days of differentiation under hypoxic conditions, the PPAR-*γ* agonist, Rosiglitazone (10 *μ*M), induced robust increases in SIRT1, PGC-1*α*, and PPAR-*α* mRNA in 1% oxygen (Figures 6(a), 6(b), and 6(c)), with little effect under 21% or 5% oxygen. Similarly, RXR-*α* mRNA under 1% oxygen was increased (Figure 6(d)), as was PPAR-*γ*, but this was not significant (data not shown). CPT-1b mRNA was increased when treated with Rosiglitazone at 1% (Figure 6(e)) as was UCP-2 (*P* < .01, data not shown), though there was no effect at 21% and 5%. Rosiglitazone treatment had no effect upon PPAR-*γ* protein, while SIRT1 protein was increased under 1% oxygen with Rosiglitazone (Figure 6(f)) and UCP-2 protein displayed an increasing trend (*P* < .06). 

**Figure 7:**
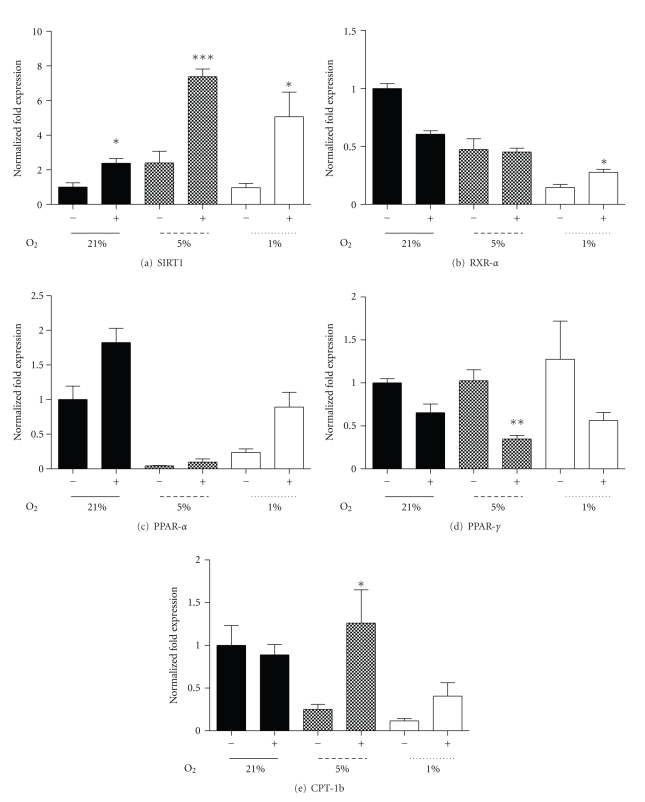
The effect of SIRT1 modulator (Resveratrol, 50 *μ*M) treatment for 24 hours, following 7 days of differentiation under relative oxygen regimes, 21%, 5%, and 1%. Fold expression of SIRT1 (a), RXR-*α* (b) PPAR-*α* (c), PPAR-*γ* (d), and CPT-1b (e) mRNA with (+) and without (−) Resveratrol. Values represent the mean of 4-5 experiments. Mean values ± SEM. The effect of Resveratrol within each oxygen regime was evaluated by Students *t*-test within oxygen regime, *P < .05, **P < .01.

**Figure 8:**
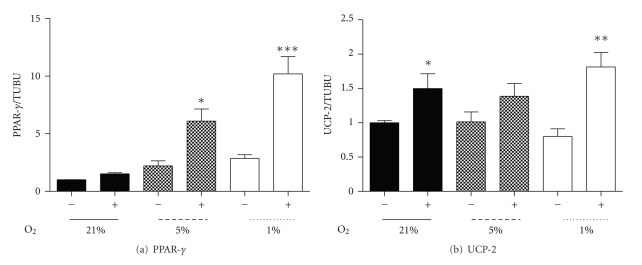
The effect of the SIRT1 modulator (Resveratrol, 50 *μ*M) treatment for 24 hours, following 7 days of differentiation under relative oxygen regimes, 21%, 5%, and 1%. Protein levels of PPAR-*γ* (a) and UCP-2 (b) with (+) and without (−) Resveratrol. Values represent the mean of 3 experiments. Mean values ± SEM. The effect of Resveratrol within each oxygen regime was evaluated by Students *t*-test within oxygen regime, *P < .05, **P < .01, ***P < .001.

Pharmacological modulators of SIRT1 activity such as Resveratrol, a natural polyphenolic compound known for its insulin sensitizing and antioxidant properties [35, 47], significantly increase SIRT1 activity *in vitro* and *in vivo* [48]. SIRT1 mRNA was significantly increased in control and hypoxic conditions (5% and 1% oxygen) with Resveratrol treatment (50 *μ*M) for 24 hours (Figure 7(a)). Resveratrol also had stimulatory effects upon RXR-*α* (Figure 7(b)) and PPAR-*α* mRNA at 1% oxygen, though the latter was not significant (Figure 7(c), *P* < .1), while no effects upon PGC-1*α* or PPAR-*β*/*δ* under hypoxia were observed. These changes occurred in association with significant decrease in PPAR-*γ* mRNA under 5% oxygen and a trend under the 1% oxygen treatment (Figure 7(d), *P* < .1). Interestingly, incubation of myotubes for 24 hours with Resveratrol, under 5% or 1% oxygen, significantly restored decreased CPT-1b mRNA expression at 5% oxygen and enhanced CPT-1b mRNA at 1% (Figure 7(e)). While Resveratrol induced a significant rise in UCP-2 mRNA under control oxygen conditions, hypoxia-induced suppression of UCP-2 mRNA was not recovered at either hypoxic treatment. Despite unaltered or reduced mRNA, both PPAR-*γ* and UCP-2 protein increased at 1% with Resveratrol (Figures 8(a) and 8(b)).

## 4. Discussion

Here, we show that chronic reduction of oxygen tensions to 5% and 1% oxygen for eight days induce decreased levels of SIRT1, PGC-1*α*, RXR-*α*, and PPAR-*α* mRNA as well as CPT-1b and UCP-2 mRNA in C_2_C_12_ muscle cells. These reductions were accompanied by unaltered PPAR-*β*/*δ* and -*γ* mRNA, but decreased SIRT1 and PGC-1*α* and elevated PPAR-*γ* protein levels. Under hypoxia, it appears that aspects of these alterations can in part be prevented, *in vitro,* through PPAR-*β*/*δ* and -*γ* agonist and SIRT1 modulator intervention. 

### 4.1. PGC-1*α* and Regulators, PPAR/RXR, Are Impaired under Hypoxia

 The muscle PGC-1*α* promoter/enhancer sequence contains a conserved peroxisome proliferator-activated receptor response element (PPRE) which binds PPAR-*β*/*δ*/RXR-*α* heterodimers in skeletal muscle [9]. Despite PPAR-*β*/*δ* mRNA and protein being unchanged in our hypoxic regimes, RXR-*α* mRNA is significantly reduced, in conjunction with reduced PGC-1*α* mRNA and protein. Previous studies have demonstrated that exposure to hypoxia reduces PPAR/RXR-binding activity and deactivates PPAR activity by reducing the availability of its obligate partner RXR-*α* in cardiac myocytes [49]. Further, this deactivation appears to be HIF-1 regulated by reducing the DNA binding of PPAR-RXR [50, 51]. In our studies, we measured the level of LOX mRNA, which is a well-established target for HIF-1*α* activity [52]. Under hypoxia, LOX mRNA was significantly elevated, supporting the notion of elevated HIF-1 activity in our culture system and is likely a primary mechanism for reduced PPAR/RXR-*α* and subsequently reduced PGC-1*α* transcription in hypoxic muscle myotubes.

### 4.2. Oxygen Tension Affects the PGC-1*α* Activator SIRT1

 C_2_C_12_ SIRT1 mRNA was unaltered in chronic muscle hypoxic culture, though SIRT1 protein was significantly depressed. This altered posttranslational modification of SIRT1 has been observed previously, where prolonged hypoxia overcame SIRT1's role in deacetylating HIF-1*α* under normoxia and resulted in activated HIF-1*α* in combination with reduced SIRT1 protein in hypoxia [53]. Prolonged hypoxia induces an altered redox potential [53, 54], and this leads to decreased SIRT1 expression through increased binding of C-terminal-binding protein-1 (CtBP-1) [55]. While there is stabilization of SIRT1 mRNA under hypoxia in the current studies, overall protein synthesis is reduced, possibly through HIF-1 actions and the phosphorylation and inhibition of eukaryotic initiation factor eIF2*α* and inactivation of eIF4F and the mTOR kinase [56]. SIRT1 is a potent NAD^+^-dependent protein deacetylase and a central regulator of PGC-1*α*/PPAR muscle oxidative process [12]. Therefore, hypoxia-induced reductions in SIRT1 levels and possible activity will further impact upon PGC-1*α* activity state and subsequent PGC-1*α*/PPAR target gene activation.

### 4.3. Hypoxia Impairs PPAR Fatty Acid Oxidation Target Genes

 Hypoxia-induced reductions in NAD^+^ correspond to reduced SIRT1 activity [55], reduced activation of PGC-1*α* and PPAR-*α* [12], and likely reduced transcription of key components of fatty acid oxidation capacity. In chronic hypoxic myotube culture, in combination with a disrupted SIRT1/PGC-1*α*/RXR/PPAR pathway, target genes CPT-1b and UCP-2 were reduced. In a similar way, hypoxic downregulation of PPAR-*α* and RXR-*α* in cardiac culture, is also associated with decreased CPT-1b and UCP-3 expression [49, 57]. The observed reductions in C_2_C_12_ CPT-1b and UCP-2, likely occur via hypoxia-induced deactivation of PPAR-*α* by reducing the availability of its obligate partner RXR. This has been reported to result in decreased DNA-binding activity of PPAR-*α*/RXR in hypoxic myocytes and subsequent target gene expression [49, 50]. Despite unaltered myotube PPAR-*β*/*δ* expression, PGC-1*α*, PPAR-*α*, and RXR-*α* transcription are suppressed, suggesting PPAR-*β*/*δ* interactions, alone, are insufficient to maintain transcription of CPT-1b and UCP-2 under chronic hypoxia.

In addition to PPAR-*β*/*δ* and -*α*, it is increasingly evident that muscle PPAR-*γ* is essential for lipid and carbohydrate metabolic homeostasis [7, 10]. PPAR-*γ* signaling activates expression of a number of oxidative and insulin-sensitizing genes including the UCPs (UCP-2 and -3), resistin, and adiponectin [58, 59]. PPAR-*γ* activity is regulated by phosphorylation status and phosphorylation of serine residue 273 in mouse embryonic fibroblasts results in a dysregulation of a large number of insulin sensitizing genes [60]. Further, the use of Rosiglitazone, which impedes aspects of PPAR-*γ* phosphorylation, improved indices of insulin sensitivity [60]. On the other hand, it is interesting to note that mutation of the other PPAR-*γ* phosphorylation site, serine 112, leads to increased transdifferentiation of C_2_C_12_ myocytes into adipocytes [61]. These results suggest a balance between phosphorylation sites may confer differential PPAR-*γ* actions. In our prolonged hypoxic conditions, despite elevated PPAR-*γ* protein, UCP-2 remained suppressed, possibly due to the reduced PGC-1*α*/RXR-*α* activity and yet to be determined hypoxia-induced alterations in PPAR-*γ* phosphorylation status and hence activity.

Further, regulation of PPAR-*γ* occurs through SIRT1. It has recently been reported that SIRT1 interacts with the transcriptional corepressor NCoR, negatively regulating PPAR-*γ* in white fat. This promotes mobilization of fatty acids, as opposed to accumulation, which occurs in situations of depressed SIRT1 [43]. If such a pathway occurs in chronically hypoxic muscle, in conjunction with the reduced SIRT1 activity, this interaction could be responsible for removing SIRT1 inhibition upon PPAR-*γ*, resulting in the observed increased PPAR-*γ* protein. Further, it is reported that PPAR-*γ* exerts a negative feedback loop effect upon SIRT1 [44]. Therefore, under hypoxia, changes in muscle PPAR-*γ* protein and activity and continual suppression of SIRT1 interactions could lead to an increased adipogenic capacity over a fatty acid oxidative state.

### 4.4. Recovery of Hypoxia-Induced Reductions in Oxidative Genes

Enhancing muscle's metabolic activity through the use of PPAR-*β*/*δ* and PPAR-*γ* agonists, has been successful in restoring skeletal mitochondrial regulation and insulin sensitivity [13, 34]. Additionally, the insulin-sensitizing effects of the polyphenol, Resveratrol, through modulation of SIRT1/PGC-1*α* activity, have been highlighted as being beneficial in tackling aspects of metabolic disease [31].

PPAR-*β*/*δ* agonists retard weight gain and promote skeletal muscle fatty acid oxidation and insulin sensitivity [45]. Under hypoxia, L165,041 treatment had minimal effects though target genes of PPAR-*β*/*δ* where activated. Similar to L6 muscle cell studies, C_2_C_12_ UCP-2 may be an additional target gene of PPAR-*β*/*δ* through L165,041 activation [39] and under hypoxia, increased UCP-2 mRNA and protein (*P* < .06) results. The continuation of this response under maintained hypoxia is to be determined.

Rosiglitazone treatment during hypoxia, on the other hand, induced robust increases in members of the SIRT1/PGC-1*α*/PPAR pathway under 1% oxygen, with little effect under 21% or 5% oxygen. These changes resulted in increased target CPT-1b and UCP-2 mRNA expression under 1% oxygen. Rosiglitazone-induced changes under hypoxia are similar to the changes reported to occur when rescuing an insulin resistant state with the related thiazolidinedione, Pioglitazone, through induction of PGC-1*α* activity [13]. Further, these data suggest that Rosiglitazone may act under hypoxia in a similar manner as reported in rescuing insulin sensitivity in alcoholic fatty liver, through increased pharmacological modulation of SIRT1-AMPK signaling [62].

Under normal oxygenation and both 5% and 1% oxygen, SRIT1 mRNA expression was increased with Resveratrol, though protein content was unaffected in the time period examined. Resveratrol specifically increases SIRT1 activity through an allosteric interaction, resulting in the increase of SIRT1 affinity for both NAD^+^ and the acetylated substrate [13, 48]. This potentially promotes an increased efficiency of SIRT1 in hypoxia as indicated by target gene activation through increased CPT-1b transcription and increased translation of UCP-2. Interestingly, however, PGC-1*α* mRNA was not recovered as would be expected. In light of differential target gene activation, Resveratrol's primary actions in maintained hypoxia may be promotion of selected fatty acid oxidation genes rather than mitochondrial biogenesis [11, 31]. Under hypoxia, it has been previously shown that Resveratrol promotes recovery of inhibitory HIF-1*α* activity and an increased PPAR-*α* expression in an oxygen/glucose deprivation neuron model [63]. Intriguingly, under 1% hypoxia, Resveratrol treatment promoted an increase in C_2_C_12_ RXR-*α*, and PPAR-*α* mRNA. A possible mechanism for this increase could be inhibition of hypoxia-mediated activation of extracellular signal-regulated kinase 1/2 and Akt, leading to a marked decrease in HIF-1*α*, as reported to occur in Resveratrol treated cancer cells [64, 65]. While treatment of hypoxia-induced changes with PPAR agonists and a SIRT1 modulator demonstrated improvements in the SIRT1/PGC-1*α*/PPAR pathway and fatty acid oxidative capacity, further work is required to fully understand the adaptation process under continued hypoxia or following reoxygenation.

## 5. Conclusions

**Figure 9:**
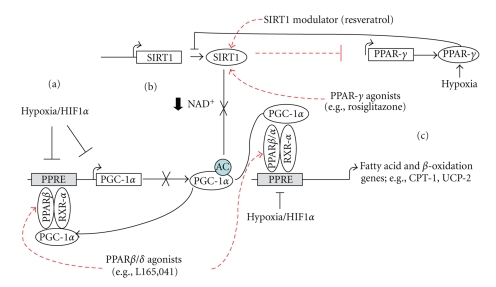
Model of how hypoxia may induce reduced myotube fatty acid oxidation gene expression. Hypoxia-induced HIF-1*α* results in impaired RXR-*α*/PPAR-*α* translation of PGC-1*α*, in combination with reduced PPAR/RXR expression (a). Further, PPAR-*γ* impaired SIRT1 translation and reduced NAD^+^ availability, resulting in reduced SIRT1 deacetylation of PGC-1*α* and activity (b). In conjunction with possible HIF-1*α* impaired PPAR-*α*/RXR-*α* PPRE binding and decreased PPAR/RXR expression, this further results in reductions of target fatty acid oxidation gene expression such as CPT-1b and UCP-2 (c). Under these reduced oxygenation conditions, it appears that aspects of the hypoxia-induced alterations can in part be prevented, *in vitro,* through PPAR-*β*/*δ* and -*γ* agonists and SIRT1 modulator intervention (dashed lines).

Our present study highlights that the expression of regulators of muscle oxidative metabolism is directly altered under reduced oxygen tension. As summarized in Figure 9, together with reductions in PPAR/RXR mRNA expression, hypoxia-induced HIF-1*α* activity impairs RXR-*α*/PPAR-*α* transcription of PGC-1*α*. In combination with hypoxia-induced PPAR-*γ*-impaired SIRT1 translation and possibly reduced NAD^+^ availability, SIRT1 deacetylation of PGC-1*α* is reduced. Further, HIF-1*α*-impaired PPAR-*α*/RXR-*α* PGC-1*α* PPRE promoter binding results in changes in downstream SIRT1/PGC-1*α* capacity. In conjunction with altered PPAR/RXR interactions, these above changes result in reductions of target fatty acid oxidation gene expression, such as CPT-1b and UCP-2. Interestingly, under these reduced oxygenation conditions, it appears that aspects of the hypoxia-induced alterations can in part be prevented, *in vitro,* through pharmacological intervention (e.g., L165,041, Rosiglitazone, and Resveratrol). These results have implications for understanding selective hypoxia adaptation and how it might impact long-term muscle fatty acid oxidative metabolism and ultimately insulin sensitivity.

## Figures and Tables

**Table 1:** Gene, accession number, forward and reverse primer sequences, and calculated efficiencies used for real-time PCR analysis.

Gene^1^	Accession no.	Forward primer	Reverse primer	Annealing temperature	Efficiency
SIRT1	NM_019812.2	5′-ATATTCCACGGTGCTGAGGT	5′-TCCAAATCCAGATCCTCCAG	59°C	96.2%
PGC-1*α*	NM_008904.2	5′-AACGATGACCCTCCTCACAC	5′-GGGTCATTTGGTGACTCTGG	59°C	99.7%
RXR-*α*	NM_011305.3	5′-GTTGCTTGTTTGCAATGGTG	5′-TGAGGAATATGGCCCAGAAG	59°C	96.3%
PPAR-*α*	NM_011144.6	5′-AACCGGAACAAATGCCAGTA	5′-CCGAATCTTTCAGGTCGTGT	59°C	94.0%
PPAR-*δ*/*β*	NM_011145.3	5′-TAGAAGCCATCCAGGACACC	5′-CCGTCTTCTTTAGCCACTGC	59°C	95.6%
PPAR-*γ*	NM_001127330.1	5′-CCAACTTCGGAATCAGCTCT	5′-CAACCATTGGGTCAGCTCTT	59°C	95.1%
CPT-1b	NM_009948.2	5′-CCCATGTGCTCCTACCAGAT	5′-CCTTGAAGAAGCGACCTTTG	59°C	95.1%
UCP-2	NM_011671.4	5′-GCCTCTGGAAAGGGACTTCT	5′-AGAAGTGAAGTGGCAAGGGA	59°C	97.7%
LOX	NM_010728.2	5′-TGCTTGATGCCAACACCCA	5′-ATGCAAATCGCCTGTGGTAGC	59°C	99.1%
RL7	NM_011291.5	5′-GGAGCTCATCTATGAGAAGGC	5′-AAGACGAAGGAGCTGCAGAAC	59°C	99.1%

^1^SIRT1, PGC-1*α*, RXR*α*, PPAR-*α*, -*β*, -*γ*, CPT-1b, UCP-2, LOX = lysyl oxidase, and RL7 = ribosomal protein L7.
